# Patient-Reported Outcomes Collection at an Urban HIV Clinic Associated With a Historically Black Medical College in the Southern United States: Qualitative Interview Study Among Patients With HIV

**DOI:** 10.2196/42888

**Published:** 2023-03-22

**Authors:** Paul E Parisot III, Facerlyn Wheeler, Kemberlee Bonnet, Peter F Rebeiro, Cassandra O Schember, Korlu McCainster, Robert L Cooper, Vladimir Berthaud, David G Schlundt, April C Pettit

**Affiliations:** 1 Department of Medicine Vanderbilt University Medical Center Nashville, TN United States; 2 Department of Family and Community Medicine Meharry Medical College Nashville, TN United States; 3 Department of Psychological Science Vanderbilt University Nashville, TN United States; 4 Division of Epidemiology Vanderbilt University Medical Center Nashville, TN United States; 5 Department of Biostatistics Vanderbilt University Medical Center Nashville, TN United States; 6 California Department of Public Health Richmond, CA United States; 7 Meharry Community Wellness Center Department of Infectious Disease Meharry Medical College Nashville, TN United States

**Keywords:** patient-reported outcomes, HIV infection, HIV continuum of care, HIV epidemic, questionnaire experience, cognitive behavioral model, implementation, vulnerable population, racial disparity

## Abstract

**Background:**

Black Americans, particularly in the southern United States, are disproportionately affected by the US HIV epidemic. Patient-reported outcome (PRO) data collection can improve patient outcomes and provide oft-overlooked data on mental health, substance use, and patient adherence to antiretroviral therapy.

**Objective:**

We piloted the use of an electronic tablet to collect PRO data on social and behavioral determinants of health among people with HIV at the Meharry Community Wellness Center, an HIV clinic affiliated with a Historically Black Medical College in Nashville, Tennessee. Our primary objective was to better understand patients’ experiences and comfort with using an electronic PRO tool through patient interviews.

**Methods:**

We enrolled 100 people with HIV in care at the Meharry Community Wellness Center consecutively to completely validate PRO tools using the Research Electronic Data Capture platform on a hand-held tablet. Using a purposive sampling strategy, we enrolled 20 of the 100 participants in an in-depth interview (IDI). Interview guide development was grounded in the cognitive-behavioral model, in which thoughts, feelings, and behaviors are interrelated. IDIs were audio recorded, transcribed, deidentified, and formatted for coding. A hierarchical coding system was developed and refined using an inductive-deductive approach.

**Results:**

Among the 100 people with HIV enrolled, the median age was 50 (IQR 42-54) years; 89% (n=89) were Black, 60% (n=60) were male, and 82% (n=82) were living below 100% of the federal poverty level in 2016. Five major interview themes emerged: overall experience, question content, sensitive topics, clinic visit impact, and future recommendations. IDI participants felt that the tablet was easy to use and that the question content was meaningful. Question content related to trauma, sexual and drug use behaviors, mental health, stigma, and discrimination elicited uncomfortable or distressing feelings in some participants. Patients expressed a strong desire to be truthful, and most would complete these surveys without compensation at future visits if offered.

**Conclusions:**

The use of an electronic tablet to complete PRO data collection was well received by this cohort of vulnerable persons in HIV care in the southern United States. Despite some discomfort related to question content, our cohort overwhelmingly believed this was a meaningful part of their medical experience and expressed a high desire for truthfulness. Future research will focus on scaling up the implementation and evaluation of PRO data collection in a contextually appropriate manner while obtaining input from providers and staff to ensure that the collected data are both applicable and actionable.

## Introduction

The southern United States is disproportionately affected by the HIV epidemic. While these states make up only 38% of the national population based on US census data, they annually account for over 50% of new HIV diagnoses, and across all regions including the southern United States, Black individuals are newly diagnosed at a rate twice that of the next closest race or ethnicity [1]. After diagnosis, Black Americans also have poorer HIV continuum of care outcomes, such as engagement in HIV care and viral suppression [2].

The Centers for Disease Control and Prevention’s National Center for HIV/AIDS, Viral Hepatitis, Sexually Transmitted Diseases, and Tuberculosis Prevention [3] has proposed addressing social and behavioral determinants of health to reduce health disparities. Many of these determinants are not routinely collected in clinical practice in a standardized way. Patient-reported outcomes (PROs), in which data are provided directly by patients without the assistance of a health care provider, can be used for improving both clinical care and research for those with and without HIV [4].

Different methods are used to collect PROs, including patient interviews, paper documents, and, with increasing frequency, they are electronically collected. A systematic review comparing electronic PRO measures to paper versions found them to be cost-effective, improve the speed and completeness of data collection, and assist with clinician decision-making by allowing the data to be immediately available [5]. In a large, urban primary care setting, the implementation of an electronic PRO interface has proven successful with high levels of satisfaction among providers, staff, and patients, suggesting a model for other clinics that see a high volume of patients [6].

Studies specific to people with HIV have shown that PROs can provide data otherwise overlooked [7] and more accurately capture data on health determinants compared to data entered into the electronic health record [8] or otherwise assessed by a health care provider [9]. PROs can provide clinicians with same-day feedback and improve provider access to and awareness of social and behavioral determinants of health data [10,11]. When completed prior to clinic visits and available directly to providers, people with HIV find PROs helpful in prioritizing and initiating discussion on sensitive topics while improving their overall satisfaction with their care [12].

The population of interest and their input should be considered when introducing new PRO tools. Other preimplementation studies of PRO design in a population of people with HIV have shown that patients desire an intuitive and secure interface with succinct and actionable information, and that obtaining such feedback could help overcome barriers to implementation [13].

Given the potential of PROs for improving patient care, prioritizing reliable and reproducible delivery that is supported by patients is necessary. Limited data exist on patient experiences with the collection of electronic PROs as part of routine care in a busy HIV clinic, particularly among majority Black populations in the southern United States. The objective of this study is to pilot and assess the acceptability of patient-reported data collection on social and behavioral determinants of health using an electronic tablet as part of routine care among people with HIV receiving care at the Meharry Community Wellness Center (MCWC), an HIV clinic associated with a Historically Black Medical College located in Nashville, Tennessee [14]. This specific clinic serves a population disproportionately affected by the US HIV epidemic, and currently, PRO data from this population are limited.

## Methods

### Study Population

We conducted a prospective study among adults with HIV receiving care at the MCWC. At the time of this study, the MCWC served as the centralized medical home for 400-500 people with HIV annually. The overall patient population was predominately Black (378/476, 79.4%), male (346/476, 72.7%), and the most frequent documented HIV risk factor was heterosexual sex (297/476, 62.4%). A significant proportion of the patients were uninsured (161/476, 33.8%), while 17.9% (85/476) were unstably housed, and 31.1% (148/476) were living below the 100% federal poverty level (FPL) in 2016.

**Figure 1:**
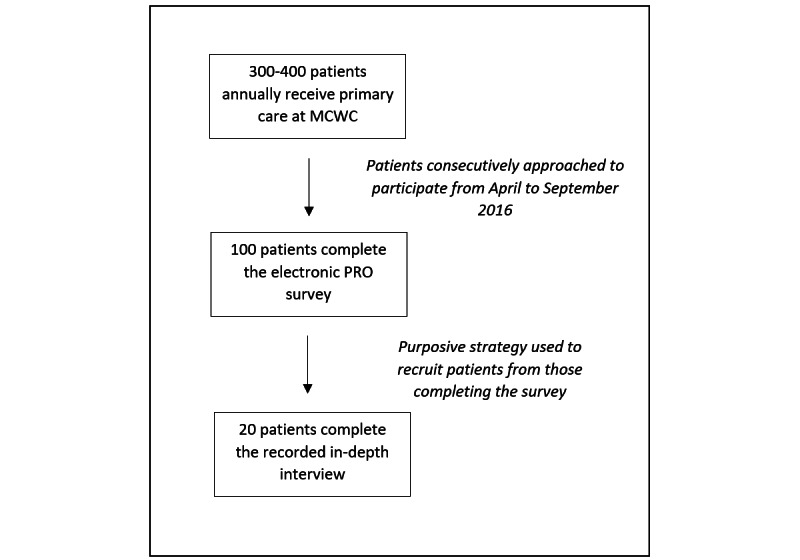
Patient recruitment and data collection. MCWC: Meharry Community Wellness Center; PRO: patient-reported outcome.

Patients were eligible if they were 18 years of age or older and presented to the MCWC for HIV-related services from April 2016 through September 2016. Patients were excluded if they could not read or speak English, as recorded by the study coordinator. We enrolled 100 consecutive participants to complete the PRO survey, and 20 of those participants were sampled using a purposive strategy to identify a set of patients with similar demographics to the overall clinic population to participate in a qualitative in-depth interview (IDI; Figure 1).

### Ethics Approval

Eligible participants provided written informed consent prior to enrollment and completion of the PRO survey. A second written informed consent was completed by the 20 individuals participating in the IDI. PRO data were collected anonymously, and IDI audio recordings were transcribed and deidentified prior to analysis. This study was approved by the institutional review boards of Meharry Medical College and Vanderbilt University Medical Center (IRB number 160017). Participants were compensated with gift cards to a local grocery store, US $15 for completion of the PRO survey, and a separate US $50 for completion of the IDI.

### Data Collection and Study Definitions

The PRO tools were all validated, web-based tools (Table 1) that were collected and stored in Research Electronic Data Capture [15]. A trained study staff member conducted an in-person interview consisting of open-ended, scripted questions related to the participant’s experience using the tablet, difficulty answering questions, feelings, emotions, or concerns while answering questions, clarity and redundancy of questions, and likes and dislikes about completing the survey. Interview guide development was grounded in the cognitive behavioral model, in which thoughts, feelings, and behaviors are interrelated [16]. Follow-up questions were asked for clarity and additional detail. Interviews were completed in a private area separate from the clinic and lasted between 10 and 30 minutes. They were audio recorded, transcribed, and formatted for coding.

**Table 1:** Patient-reported outcome data collection instruments.

Data collection instrument	Definition
Alcohol Use Disorders Identification Test (AUDIT-C) [17]	Alcohol use disorder in the past 12 months defined as AUDIT-C score >4 for men and >3 for women; binge drinking defined as ≥6 drinks on 1 occasion
Modified Alcohol, Smoking, Substance Involvement Screening Test [18]	Substance use during the previous 3 months categorized as present or absent
Tobacco Use National Health Interview Survey [19]	Smoking status defined as never smoker, former smoker, and current smoker
Patient Health Questionnaire-4 [20]	At-risk for depression or anxiety in the past 2 weeks defined by scores of >3 from the depression or anxiety subscale items
Perceived Stress Scale-10 [21]	Continuous score with higher scores indicating higher perceived stress in the past month
Everyday Discrimination Scale [22]	Continuous score with higher scores indicating higher perceived discrimination; scored on a 6-point scale ranging from “almost every day” discrimination to “never”
HIV Risk Behavior Scale (Sex) [23]	Continuous score with higher scores indicating increased high-risk sexual behaviors in the past month
Childhood Trauma Questionnaire [24]	Continuous score with higher scores indicating increased childhood trauma
Acceptance and Action Questionnaire II [25]	Continuous score representing current psychological flexibility with higher scores representing inflexibility
Brief HIV Stigma Scale-10 [26]	Continuous score with higher scores indicating higher perceived HIV stigma
AIDS Clinical Trials Group Adherence Questionnaire [27]	Primarily assesses ART^a^ adherence by 4-day patient reported recall
Life Events Checklist for DSM-5^b^ [28]	A checklist of events that place one at risk for PTSD,^c^ no formal scoring protocol

^a^ART: antiretroviral therapy.

^b^DSM-5: Diagnostic and Statistical Manual of Mental Disorders, Fifth Edition.

^c^PTSD: posttraumatic stress disorder.

Additional covariates of interest were abstracted from the electronic health record and included age at enrollment (years), self-reported gender identity (male, female, transgender man, and transgender woman), self-reported race or ethnicity (Black non-Hispanic, White non-Hispanic, and other or unknown), HIV transmission risk factors (heterosexual contact, male-male sexual contact, injection drug use, and other or unknown), CD4+ lymphocyte count and viral load within 120 days prior to and up to 7 days after enrollment, income relative to the FPL [29], and housing status (stable or permanent, temporary, unstable, and missing).

### Statistical Analysis

We summarized categorical variables using counts and proportions and continuous variables using medians and IQRs. IDI data coding, analysis, and reporting followed the Consolidated Criteria for Reporting Qualitative Research guidelines [30]. A hierarchical coding system was developed based on interview guide questions and a preliminary review of transcripts using an inductive-deductive approach [31]. The coding system was organized into 5 major themes: overall experience, specific questions, sensitive topics, clinic visit impact, and future recommendations.

Each response was treated as a separate quote and assigned up to 5 different codes. Two trained coders independently coded the quotes and established interrater consensus when differences in coding occurred. Quotes were sorted by category, and frequency distribution tables were created to identify higher-order themes.

## Results

### Quantitative Data

Among the 100 participants, the median age was 50 years (IQR 42-54), 60% (n=60) were male, 89% (n=89) were Black, and 59% (n=59) reported heterosexual contact as their HIV transmission risk factor. The majority (n=82, 82%) were living below the FPL, while about 25% (n=25) were living in temporary or unstable housing (Table 2).

Substance use was prevalent; a total of 26% (n=26) reported binge drinking in the previous 12 months, 39% (n=39) used marijuana within the past 3 months, and 34% (n=34) reported cocaine use within the prior 3 months. Anxiety was reported by 41% (n=41) and depression by 55% (n=55). Half of those surveyed (n=50, 50%) reported <95% antiretroviral therapy (ART) adherence in the 4 days preceding their visit (Table 3).

**Table 2:** Demographic and clinical characteristics of the study population (Meharry Community Wellness Clinic, 2016; N=100).

Characteristic		Values
Age (years), median (IQR)		50 (42-54)
**Gender, n (%)**		
	Male	60 (60)
	Female	37 (37)
	Transgender woman	3 (3)
**Race or ethnicity, n (%)**		
	Black, non-Hispanic	89 (89)
	White, non-Hispanic	7 (7)
	Other or unknown	4 (4)
**HIV transmission risk, n (%)**		
	Heterosexual contact	59 (59)
	Male-to-male sexual contact	22 (22)
	Injection drug use	14 (14)
	Other or unknown	5 (5)
CD4+ lymphocyte count (cells/µL), median (IQR)		541 (337-805)
HIV-1 RNA (log_10_ copies/mL), median (IQR)		Undetectable (Undetectable to 45)
**Income relative to federal poverty level (FPL), n (%)**		
	<100% FPL	82 (82)
	100%-137% FPL	8 (8)
	138%-199% FPL	3 (3)
	≥200% FPL	3 (3)
	Missing	4 (4)
**Housing status, n (%)**		
	Stable or permanent	71 (71)
	Temporary	24 (24)
	Unstable	1 (1)
	Missing	4 (4)

**Table 3:** Patient-reported outcomes of the study population (Meharry Community Wellness Clinic, 2016; N=100; scoring criteria shown summarized in Table 1).

Outcomes		Values
**Binge drinking status, n (%)**		
	No reported binge drinking	74 (74)
	Binge drinking ≥1 occasion	26 (26)
**Hazardous drinking** **, n (%)**		
	No	81 (81)
	Yes	19 (19)
**Tobacco use** **, n (%)**		
	Current	52 (52)
	Former	9 (9)
	Never	32 (32)
	Don’t know or missing	7 (7)
**Marijuana use in the past 3 months, n (%)**		
	No use	61 (61)
	Any use	39 (39)
**Cocaine use in the past 3 months, n (%)**		
	No use	66 (66)
	Any use	34 (34)
**Anxiety status in the past 2 weeks, n (%)**		
	Not anxious	57 (57)
	Anxious	41 (41)
	Missing	2 (2)
**Depression status in the past 2 weeks, n (%)**		
	Not depressed	43 (43)
	Depressed	55 (55)
	Missing	2 (2)
**Anxiety and depression in the past 2 weeks** **, n (%)**		
	Neither anxious nor depressed	40 (40)
	Both anxious and depressed	58 (58)
Perceived Stress Scale score, median (IQR)		19 (16-23.5)
Everyday Discrimination Scale score, median (IQR)		9 (5.5-13)
HIV Risk Behavior Scale score, median (IQR)		10 (7-13)
**Life Events Checklist** **, median (IQR)**		
	Happened to me	3.5 (1-6)
	Witnessed	0 (0-2)
Acceptance and Action Questionnaire score, median (IQR)		17 (10.5-29)
Brief HIV Stigma Scale score, median (IQR)		21 (17-26)
**ART^a^ adherence status in the past 4 days, n (%)**		
	<95% adherent	50 (50)
	≥95% adherent	45 (45)

^a^ART: antiretroviral therapy.

### Qualitative Data

Five main themes emerged, which are summarized with representative quotes below with additional representative quotes included in Textbox 1.

**Major interview themes and additional representative quotes.**

**Overall experience using the electronic tablet**

- *One thing you can see things and you can go forward and do that quicker than a pencil.*
- *It was something different knowing that I am computer illiterate, but it was easy.*
- *Yes, certain questions that I wouldn’t feel comfortable answering out loud I was able to answer on the iPad.*
- *Because it throw me off for the next answer. I might be beginning to open up on suggestions of things that I’m going through in life. Then I get all scrambled on the iPad. Then I’ll just want to get through the mess.*
- *No, I’m a paper person; I’m just used to the pen and paper.*
- *I think I took about 30 if it took a little more, but it was worth answering. It was worth it.*

**Thoughts and recollections regarding specific questions**

- *I remember that it was basically focusing on adhering to your HIV regimen, and if there’s any reasons that you don’t take the medication, why do you miss a dose?*
- *Lot of them was easy to answer because I’ve had HIV for a while now.*
- *I guess the factual ones like how many times have you done this or what’s your sexual history or if you smoke or drink. Those are easy to answer. Yes or No. I guess those are pretty simple, straightforward.*
- *No. They were centered around more of what the hell was I thinking back then and how did I get to this point now? It’s just a reflective part where I’m thinking about what was on my mind, but I’m just grateful that my mind frame has changed, so it was one of those reflective things, also.*

**Feelings regarding sensitive topics**

- *Feeling like life is out of control, feeling like my self-worth. On one hand I’ve come a long way but on the other hand, too I think looking at them I still probably have a way to go or still probably have a lot of bad days too so just trying to get an honest answer made me do some self-reflection so they were kind of tough.*
- *The traumatic events brought up bad memories because I’ve found people, you know, my sister-in-law and my brother both dead, and one was very recent, and I tried to get over that.*
- *Basically, I was feeling a little embarrassed, maybe? Because of my being promiscuous...*
- *Yes. Answering those questions, it did open up a door thinking back to the past and it made me feel kind of nauseated and sick to my stomach because it made me think back to the fact of how I contracted HIV, that my boyfriend at the time had known he was positive since 1990 and he didn’t disclose that to me, so it opened that door back up again answering those questions.*

**Reflections on how this exercise affected perception of clinical visits**

- *It teach me to be honest when I come to the clinic about whatever is happening with my life...*
- *It just made me more aware that I need to continue to be prompt with my appointments, how important it is and was for me to continue to take my medicines as prescribed and keep trying to live a healthy life to my best ability, living with HIV.*
- *By answering these questions, they learn more about what I’m going through right now, and this right here helped me out…*
- *That’s the whole point of the survey and then too not only just me but it might could help someone that’s in a similar situation too. That’s why even though it was for the compensation, I wanted to take the effort and really think about and give an accurate answer and give someone because that helps give back.*

**Recommendations for improvements**

- *The only thing that I could see, in my personal opinion, pertaining to the questions was it didn’t inquire anything pertaining to how a person living with HIV or AIDS, family-wise. It didn’t include anything about the children, if those of us who have children, how we were being treated or how we interacted with our children and family members.*
- *They need to know there’s a lot of us out here that are trapped within our lifestyles and trying to find a way to get out of it.*
- *How am I taking care of myself? Am I taking my meds? Are you sleeping well? What kind of feelings do I have when I wake up?; I didn’t hear a lot about a support system.*

#### Overall Experience Using the Electronic Tablet

Initial expectations about ability to use the electronic tablet were variable; a total of 25% (n=5) cited prior experience, while another 25% (n=5) described no or minimal similar experiences, the other remaining individuals did not comment on prior experience. Of those with no or minimal prior experience, a total of 40% (n=2) still felt confident with their abilities. Most individuals (n=16, 80%) regardless of prior experience valued the usability of the tablet, specifically they appreciated the speed (10-30 minutes), ease of correcting mistakes compared to a paper version, the capacity to adjust the screen, or other navigation features. A minority required technical assistance to use the device (n=2, 10%) or preferred a paper over an electronic version (n=2, 10%).

It was pretty straight forward and I think that’s what I appreciate. You can just push the buttons and click and keep moving. Even for people who don’t use iPads it’s pretty easy to learn.

#### Thoughts and Recollections Regarding Specific Questions

There was mixed feedback about using the electronic tablet to answer questions pertaining to sensitive information. While some participants (n=13, 65%) were not worried about others seeing their responses, others (n=5, 25%) did express some concerns about data confidentiality, and the remainder (n=2, 10%) was unsure. A minority (n=1, 5%) felt that the electronic tablet removed humanity from the questions.

It would be more comfortable using the iPad because having an interview face to face, someone may feel a little timid, and I have in the past… thinking maybe someone’s judging me because of the answers I’m giving, but today I don’t feel that way.

Participants identified the most difficult questions as those regarding past behaviors or experiences such as sexual behavior, drug use, sexual assault, mental health, discrimination regarding their HIV status, and fear of law enforcement. Questions about social support systems and treatment adherence were easier for participants to answer in the setting of HIV status acceptance and participation in long-term HIV treatment.

I couldn’t say any question was hard for me because ... Now, today I’ve come to terms that I am HIV positive and it’s a part of who I am.

Many participants (n=11, 55%) felt that questions pertaining to substance use or missed medication were repetitive.

…but a lot of the questions were the same questions, but they were reworded and in my thinking of when they do that on any survey or any test, they just looking to see if you’re paying attention or are you just marking anything.

#### Feelings Regarding Sensitive Topics

In discussing emotional reactions to survey topics, feelings discussed included regret, embarrassment, and stigmatization, specifically with respect to questions regarding past trauma, substance use, or sexual behavior.

I immediately felt put on the spot based on my previous past behavior, how I acted out. I know I’m not that way anymore, but the implications in the questions to me were, ‘Well this is who you still are.’...

Some participants (n=7, 35%) worried about the possibility of unwanted HIV status disclosure or behavioral history. Those not worried about their responses mentioned protection through privacy policies, trust in their doctors, and acceptance of their HIV status.

My instant gut reaction is I felt intruded upon, because I thought somebody’s wanting to document me, not a person with HIV… That to me, throws up flags… It made me uncomfortable for a brief minute.

All participants reported a desire to give honest answers, with reasons for honesty including self-accountability, helping other people with HIV, and building an honest relationship with their HIV health care provider. They discussed living with HIV and the importance of having someone close to talk to, as well as the recognition of grief, gratitude, religion or spirituality, acceptance, hope, unity, and community.

You have to take care of yourself. You can’t blame it on nobody else. You got it, you got it, that’s it, so I’ve always taken care of myself…Just to have people you can talk to, it means a hell of a lot. I’m not out there on the streets. I just keep trying to live day to day.

#### Reflections on How This Exercise Affected the Perception of Clinical Visits

Many participants (n=9, 45%) viewed the survey as meaningfully improving their overall clinic experience, while no participants felt it negatively affected their experience. Some felt it provided accountability regarding their health and attending appointments (n=5, 25%). Most participants (n=16, 80%) felt their answers were important for the improvement of their health and to provide their medical providers with information that otherwise may not have been discussed.

It makes me want to be more open and just freely tell my doctor more things about me with the expectation he’ll be able to treat me, medically, better.

Some participants perceived the survey content as important for improving the quality of care for others beyond themselves (n=6, 30%). Specifically, they thought these questions might lead to a more in-depth understanding of the impact of HIV on people’s lives. Most participants (n=17, 85%) were willing to complete this or similar surveys at future visits, regardless of compensation.

I wouldn’t mind and care. It wouldn’t bother me… Sometime with me, talking about it relieves so much pressure, because that way, I can get out stuff that I can’t tell nobody else…

#### Recommendations for Improvement

Participants recommended adding educational programming on medication adherence and side effects (n=7, 35%). Many participants (n=11, 55%) also felt their health care providers needed to know more about their mental health, coping strategies, support systems, or quality of life. Specifically, participants shared a common sentiment that health care providers should have the information and resources to support people with HIV dealing with challenging circumstances such as housing instability, abandonment, domestic life stresses, or food insecurity.

## Discussion

### Principal Findings

In this population of adults with HIV in care at an HIV clinic associated with a Historically Black Medical College in the southern United States, PROs were deemed valuable for improving patient medical care. Previous studies of electronic PRO collection among people with HIV have shown value in effectively collecting social and behavioral determinants of health data [7-11]; however, this work adds to the previous literature by demonstrating that results extend to a vulnerable population of people with HIV in the southern United States and by providing rich qualitative data about the complexities of completing PRO data collection.

Most participants expressed a positive experience using the electronic tablet, even among those with limited technology experience. A consortium evaluating electronic PRO collection for clinical trials recommends preintervention comprehensive training for both staff and participants [32]. Though such intensive training may not be feasible for a busy HIV clinic, providing staff training protocols and participant tutorials may help ease implementation for staff and reach participants with limited technology experience to improve response rates.

Previous studies have shown discrepancies and underreporting of mental health and substance use data when collected by providers as opposed to PRO data [8,9]. This may suggest that the provider-collected data may be subject to social desirability bias, which could be mitigated through PRO collection. Despite the strong emotional responses elicited by questions on these topics among our study population, the overwhelming majority reported a conscious effort to be detailed and honest in their responses, as they believed this was important to improve health care experiences for themselves and others. These strong emotional responses highlight the critical need to use a trauma-informed care lens in HIV care. Trauma-informed approaches require that individuals at all levels of an organization realize the prevalence and health impacts of trauma, recognize the signs and symptoms of trauma, respond to trauma experiences, and resist retraumatization [33]. Future PRO implementation efforts should incorporate these approaches to mitigate the impact of trauma on people with HIV.

Alarmingly, half of the surveyed participants reported <95% recent ART adherence. Participants specifically requested education about the medications they were taking and potential side effects to help improve their ART adherence. There is limited research about directly including educational materials in PRO surveys. However, PRO data collection via electronic tablets could provide an opportunity to assess the educational needs of people with HIV and deliver educational materials. Further work is needed to determine the optimal ways to screen for educational needs and identify methods of educational material delivery among people with HIV.

Recommendations for additional questions included those assessing social support systems, family life, coping with HIV, food insecurity, and unstable housing, which have all been associated with poor ART adherence and unsuppressed viremia [34,35]. While these social determinants of health may pose a challenge to address in a 15- to 20-minute clinic visit, brief screenings could identify patients that may benefit from additional social work and connections to relevant community resources. Expanding PRO surveys to include topics important to patients requires educating the providers on appropriate responses and available resources.

### Limitations and Future Considerations

Our study has several limitations. First, this was a single-site study conducted at 1 HIV clinic in the southern United States that serves a predominantly Black population. While the results of this study may not be generalizable to other subpopulations of people with HIV residing in other areas of the United States, understanding PRO data collection among this vulnerable population disproportionately affected by the US HIV epidemic is critical. Second, we excluded participants who were unable to read or speak English. Previous studies have found high acceptability of PRO data collection among Spanish-speaking people with HIV [36], and assessment of the most frequent primary languages in this clinic population will be important moving forward. Additionally, Research Electronic Data Capture has a “Text-to-Speech” function that can be explored for English-speaking people with HIV who are unable to read or have significant visual deficits in future studies [37].

Clinic workflow impacts are also a critical consequence to consider in PRO implementation. The set of tools in this study required 10-30 minutes for completion, which is not currently built into routine visits and could slow clinic workflow. Other studies demonstrated that adjusting appointment times, creating private areas away from other patients to decrease distractions, or allowing patients time between intake and medical visits helped overcome logistical challenges [9]. Additionally, the set of tools could be shortened to include only the most critical elements, or the frequency of the data collection could be lengthened to mitigate its impact on clinic workflow. Future preimplementation work in this area will be important prior to further scaling up PRO collection in similar settings.

While considering the impact on workflow of completing such surveys, one must also consider the potential additional time required for a medical provider to review and act on the data. Providers have previously stressed the need for education on the appropriate usage of the information collected by PROs [11]. One study found that when evidence of mental health or substance use disorders was found through a PRO, the providers who received simultaneous clinical recommendations had an increased likelihood of developing action plans to address the concern, emphasizing the importance of providing a predetermined recommendation [38]. Preparing providers for interpreting and acting on this additional data may ease such burdens.

Our study did not elicit provider or staff input regarding the feasibility of implementation. A similar study found variability between patients and providers in the perceived importance of several PRO domains [39]. Provider feedback may provide a better understanding of these possible discrepancies and how they may be addressed before implementing a routine PRO survey.

Another possible limitation lies in a potential selection bias in our recruitment process. All participants received compensation, which may have led to a volunteer bias. As such, our study population was overrepresented by individuals living below the FPL compared to the overall clinic population (82/100, 82% vs 148/476, 31.1%). However, other demographic and clinical characteristics of our study population were similar to that of the overall clinic population with respect to age, gender, race, and HIV risk factor.

### Conclusions

Standardized, routine detection of social and behavioral determinants of health is imperative for meeting the National Center for HIV/AIDS, Viral Hepatitis, Sexually Transmitted Diseases, and Tuberculosis Prevention’s goal to reduce health disparities among people with HIV [3]. Electronic PRO collection was well-received even among those with limited prior technology experience and considered personally meaningful for this vulnerable population of people with HIV in the southern United States. Though participants expressed some discomfort with question content, there was a strong desire to provide truthful answers regarding social and behavioral determinants of health and a willingness to complete similar PRO data in the future. This study will guide future research focused on scaling up the implementation with the inclusion of clinical staff input to ensure PRO data are gathered in a contextually appropriate and actionable manner.

## Abbreviations

ART: antiretroviral therapy
FPL: federal poverty level
IDI: in-depth interview
MCWC: Meharry Community Wellness Center
PRO: patient-reported outcome

## Data Availability

The data sets generated and analyzed during this study are available from the corresponding author on reasonable request.
